# Positronenemissionstomographie-negative hämophagozytische Lymphohistiozytose bei zwei Patienten mit aggressivem B‑Zell-Lymphom

**DOI:** 10.1007/s00108-025-01957-7

**Published:** 2025-08-04

**Authors:** Pascal Migaud, Kai Hosmann, Markus Müller, Barbara Ingold-Heppner, Hartmut Stocker

**Affiliations:** 1 Klinik für Infektiologie, St. Joseph Krankenhaus Berlin-Tempelhof, Wüsthoffstraße 15, 12101 Berlin, Deutschland; 2 Charité – Universitätsmedizin Berlin, Berlin, Deutschland; 3 Klinik für Gastroenterologie und Onkologie, St. Joseph Krankenhaus Berlin-Tempelhof, Berlin, Deutschland; 4 Institut für Histopathologie, DRK Kliniken Berlin Westend, Berlin, Deutschland

**Keywords:** Positronenemissionstomographie/Computertomographie, ^18^F‑Fluordesoxyglukose, Diffuses großzelliges B‑Zell-Lymphom, Hämophagozytische Lymphohistiozytose/Biopsie, Knochenmarkdiagnostik, Etoposid, Positron emission tomography computed tomography, fluorodeoxyglucose F18, Lymphoma, large B‑cell, diffuse, Lymphohistiocytosis, hemophagocytic/biopsy, Bone marrow examination, Etoposide

## Abstract

**Einleitung:**

Die hämophagozytische Lymphohistiozytose (HLH) ist ein lebensbedrohliches hyperinflammatorisches Syndrom, das durch verschiedene Erkrankungen ausgelöst wird, wobei Lymphome zu den wichtigsten Triggern gehören. Die Positronenemissionstomographie/Computertomographie (PET/CT) gilt als wichtiges Instrument bei der Suche nach der Genese einer HLH.

**Fallbeschreibungen:**

Wir stellen zwei Fälle von HLH vor, die durch ein diffuses großzelliges B‑Zell-Lymphom („diffuse large B‑cell lymphoma“ [DLBCL]) ausgelöst wurden und bei denen die ^18^F‑Fluordesoxyglukose(^18^F‑FDG)-PET/CT keine abnorme FDG-Aufnahme zeigte. *Erster Fall:* Ein 72-jähriger Mann stellte sich mit intermittierendem Fieber und B‑Symptomen vor. Eine umfassende Untersuchung, einschließlich PET/CT, ergab keine Erklärung für die Verschlechterung der klinischen Situation. Es wurde eine HLH mit 6 von 8 HLH-Kriterien, 99 %iger Wahrscheinlichkeit im HScore und einem positiven Optimized-HLH-Inflammatory(OHI)-Index diagnostiziert. Nach Durchführung einer Knochenmarkpunktion und Hautbiopsie wurde eine HLH-gerichtete Therapie mit Etoposid und Dexamethason eingeleitet. Der Patient verstarb unerwartet. Die postmortale histopathologische Untersuchung ergab ein DLBCL im Knochenmark, eine zusätzliche Infiltration des peripankreatischen Fettgewebes und des Herzmuskels sowie intravaskuläre Manifestationen in der Leber. *Zweiter Fall:* Eine 58-jährige Frau stellte sich mit einer Vorgeschichte von Fieber und erhöhten Entzündungsmarkern vor. Eine umfassende diagnostische Untersuchung einschließlich PET/CT ergab keine auffälligen Befunde. Die Diagnose HLH wurde gestellt (7 von 8 HLH-Kriterien, HScore > 99 %, positiver OHI-Index). Die Knochenmark- und Leberpunktion ergab eine Hämophagozytose ohne Hinweise auf ein Lymphom. Eine Biopsie der makroskopisch normalen Haut ergab schließlich ein intravaskuläres DLBCL. Die Therapie mit Rituximab, Cyclophosphamid, Doxorubicin, Vincristin und Prednisolon (R-CHOP) und hoch dosiertem Methotrexat führte zu einer vollständigen Remission.

**Schlussfolgerung:**

Die HLH ist eine seltene Komplikation eines DLBCL. In der PET/CT können Hinweise auf ein zugrunde liegendes Lymphom fehlen. Umfangreiche Biopsien, auch aus makroskopisch unauffälligen Strukturen, können zur Diagnose eines Lymphoms führen. Anhaltendes Fieber sollte Anlass zur Abklärung einer möglichen HLH geben. Sobald die Diagnose HLH feststeht, ist eine rasche Abklärung mit frühzeitiger Knochenmarkpunktion sowie Biopsie der Haut und möglicherweise weiterer Strukturen der Schlüssel zur Diagnosefindung.

## Abstract (en)

**Introduction:**

Hemophagocytic lymphohistiocytosis (HLH) is a life-threatening hyperinflammatory syndrome caused by various diseases, with lymphoma being one of the most important triggers. Positron emission tomography/computed tomography (PET/CT) is considered an important tool in establishing the etiology of HLH.

**Case descriptions:**

We present two cases of HLH caused by diffuse large B‑cell lymphoma (DLBCL) in which 18F-fluorodeoxyglucose (18F-FDG) PET/CT showed no abnormal FDG uptake. *Case 1*: A 72-year-old man presented with intermittent fever and B symptoms. A comprehensive workup, including PET/CT, were inconclusive. HLH was diagnosed with 6 of 8 HLH criteria, a 99% probability in the H‑score, and a positive Optimized HLH Inflammatory (OHI) index. After performing bone marrow aspiration and skin biopsy, HLH-directed therapy with etoposide and dexamethasone was initiated. The patient died unexpectedly. Postmortem histopathologic examination revealed DLBCL in the bone marrow, and additional infiltration of the peripancreatic adipose tissue, and myocardium, and intravascular manifestations in the liver. *Case 2*: A 58-year-old woman presented with a history of fever and elevated inflammatory markers. Comprehensive diagnostic workup including PET/CT revealed no abnormal findings. The diagnosis of HLH was made (7 of 8 HLH criteria, H‑score > 99%, positive OHI index). Bone marrow and liver aspiration revealed hemophagocytosis without evidence of lymphoma. A biopsy of macroscopically normal skin finally revealed intravascular DLBCL. Treatment with rituximab, cyclophosphamide, doxorubicin, vincristine, and prednisolone (R-CHOP) and high-dose methotrexate resulted in complete remission.

**Conclusion:**

HLH is a rare complication of DLBCL. PET/CT may show no evidence of underlying lymphoma. Extensive biopsies, including macroscopically unremarkable structures, may lead to the diagnosis of lymphoma. Persistent fever should be a reason to investigate possible HLH. Once the diagnosis of HLH is established, a rapid work-up with early bone marrow aspiration, skin biopsy and possibly other structures is key for diagnosis.

## Hintergrund

Die hämophagozytische Lymphohistiozytose (HLH) ist ein seltenes, lebensbedrohliches hyperinflammatorisches, hyperferritinämisches Syndrom, das durch einen Zytokinsturm, eine übermäßige T‑Zell-Proliferation und eine unkontrollierte Makrophagenaktivierung gekennzeichnet ist. Die wichtigsten klinischen Befunde sind Fieber, Hepato- und/oder Splenomegalie und Panzytopenie. Die primäre (genetische) Form betrifft vor allem Säuglinge, während die sekundäre HLH in jedem Alter auftritt und durch Infektionen, rheumatologische Erkrankungen oder maligne Erkrankungen (M-HLH), insbesondere Lymphome, ausgelöst wird [1]. Die Diagnose der HLH, die häufig mit einer Sepsis verwechselt wird, und insbesondere die Identifizierung des Auslösers stellen eine klinische Herausforderung dar. Die Verzögerung der Diagnose ist mitverantwortlich für die hohe Sterblichkeitsrate von bis zu 80 % bei M‑HLH [2, 3]. Die HLH-2004-Kriterien, der HScore und der neuere Optimized-HLH-Inflammatory(OHI)-Index, der speziell für M‑HLH verwendet wird, sind gut etablierte Diagnoseinstrumente. Für den HScore wird in der Regel ein Grenzwert von 169 Punkten zur Diagnose einer HLH gewählt. Dieser Wert geht mit einer Sensitivität von 93 % und einer Spezifität von 86 % einher und erlaubt eine korrekte Klassifikation der Patienten in 90 % der Fälle [4, 5].

Für die hämophagozytische Lymphohistiozytose gibt es gut etablierte Diagnoseinstrumente

Die diagnostische Untersuchung zur Suche nach der auslösenden Grunderkrankung beinhaltet die Biopsie von Knochenmark, Lymphknoten und, falls angezeigt, Haut, Milz oder Leber. Die ^18^F‑Fluordesoxyglukose-Positronenemissionstomographie/Computertomographie (^18^F‑FDG-PET/CT) zu Beginn des Diagnosealgorithmus ist ein weiterer wichtiger Bestandteil [6, 7]. Hier stellen wir die Fälle von zwei Patienten mit Positronenemissionstomographie/Computertomographie(PET/CT)-negativer M‑HLH vor.

## Fall 1

Ein 72-jähriger Mann mit bekannter Autoimmunhepatitis unter niedrig dosiertem Prednisolon (2,5 mg/Tag) und Azathioprin (75 mg/Tag) wurde mit einer 4‑wöchigen Vorgeschichte von intermittierendem Fieber, Gewichtsverlust, Nachtschweiß und Schüttelfrost in unser Krankenhaus überwiesen. Die Entzündungsparameter waren massiv erhöht (Patientencharakteristika in Tab. 1).

**Tab. 1:** Patientencharakteristika

**Fall**	1	2
**Geschlecht**	Männlich	Weiblich
**Alter (Jahre)**	72	58
**HLH-Kriterien**		
*Fieber (>* *38,5* *°C; >* *6 Tage)*	+	+
*Löslicher Interleukin-2-Rezeptor (>* *2400* *IU/µl)*	50.276	11.148
*Zytopenien (mindestens* *2)*	+	+
Neutrophile (< 1000/µl)	5530	2700
Hämoglobin (< 9,0 g/dl)	7,7	6,4
Thrombozyten (< 100.000/µl)	76.000	22.000
*Hepatomegalie/Splenomegalie*	+	+
*Hypertriglyzeridämie/Hypofibrinogenämie*	–	+
Triglyzeride (> 3 mmol/l)	2,55	4,24
Fibrinogen (< 1,5 g/l)	3,61	1,1
*Ferritin (>* *500* *µg/l)*	8427	7976
*Hämophagozytose im Knochenmark*	+	+
*Niedrige oder fehlende NK-Zell-Aktivität*	n. b.	n. b.
*Gesamtzahl der erfüllten Kriterien*	6/8	7/8
**HScore (absolut/Wahrscheinlichkeit)**	244/99 %	255/99 %
**HLH-gerichtete Therapie**	Dexamethason + Etoposid	Dexamethason + Etoposid

*+* vorhanden, *−* nicht vorhanden, *HLA* hämophagozytische Lymphohistiozytose, *n.* *b.* nicht bestimmt, *NK-Zelle* natürliche Killerzelle

Umfangreiche Untersuchungen, einschließlich Blutkulturen, Röntgenaufnahmen und konventioneller Computertomographie(CT)-Scans, transösophagealer Echokardiographie und schließlich PET/CT, ergaben keine relevanten Befunde. Aufgrund der klinischen Verschlechterung wurde eine Verlegung in unser Krankenhaus veranlasst.

**Abb. 1:**
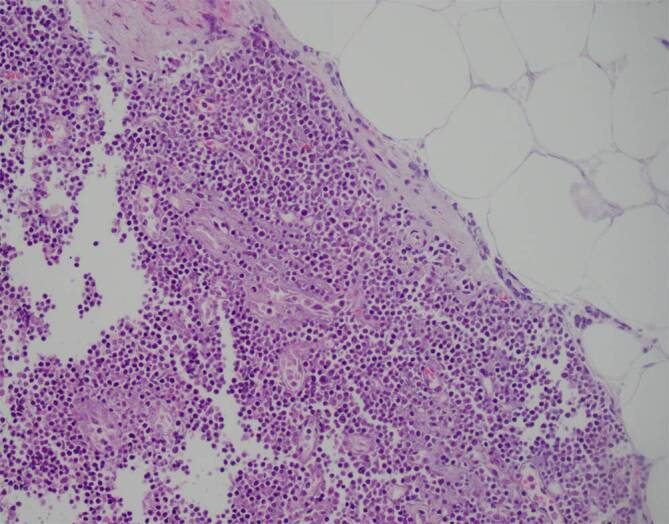
Peripankreatisches Fettgewebe mit Lymphominfiltraten

**Abb. 2:**
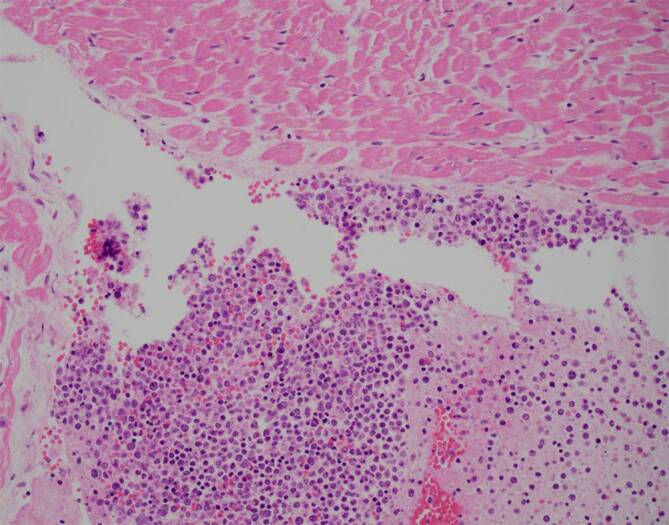
Myokard mit Lymphominfiltraten

Wir diagnostizierten eine HLH bei 6/8 positiven HLH-2004-Kriterien, 244 Punkten im HScore (Wahrscheinlichkeit 99 %) und einem positiven OHI-Index mit einem Ferritinwert von 8427 ng/ml und einem Wert des löslichen Interleukin-2-Rezeptors von 50.276 U/ml. Nach Knochenmarkpunktion und Hautbiopsien leiteten wir noch am selben Tag eine M‑HLH-Therapie mit Etoposid (100 mg/m^2^) und Dexamethason (10 mg/m^2^) ein. Am nächsten Tag verstarb der Patient unerwartet. Die Knochenmarkpunktion ergab ein diffuses großzelliges B‑Zell-Lymphom („diffuse large B‑cell lymphoma“ [DLBCL]) vom Nicht-Keimzentrum-Zell-Typ (Koexpression von CD20 und c‑Myc, jedoch nicht von BCL2 und BCL6) mit einer hohen Wachstumsfraktion von 50 % und einer Hämophagozytose. Die Autopsie ergab außerdem eine Infiltration des Lymphoms in das peripankreatische Fettgewebe (Abb. 1) und Myokard (Abb. 2) sowie eine intravaskuläre Manifestation in der Leber. Als Todesursache vermuteten wir eine Arrhythmie im Zusammenhang mit der Myokardinfiltration.

## Fall 2

Eine 58-jährige, zuvor gesunde Frau stellte sich mit seit etwa einem Monat anhaltendem Fieber in einem anderen Krankenhaus vor. Die mikrobiologische und konventionelle radiologische Diagnostik erbrachte keinen Hinweis auf eine Ursache. Mehrere Behandlungen mit Breitspektrumantibiotika führten zu keiner Besserung. Die Patientin wurde zur weiteren Abklärung und Behandlung in unser Krankenhaus verlegt.

**Abb. 3:**
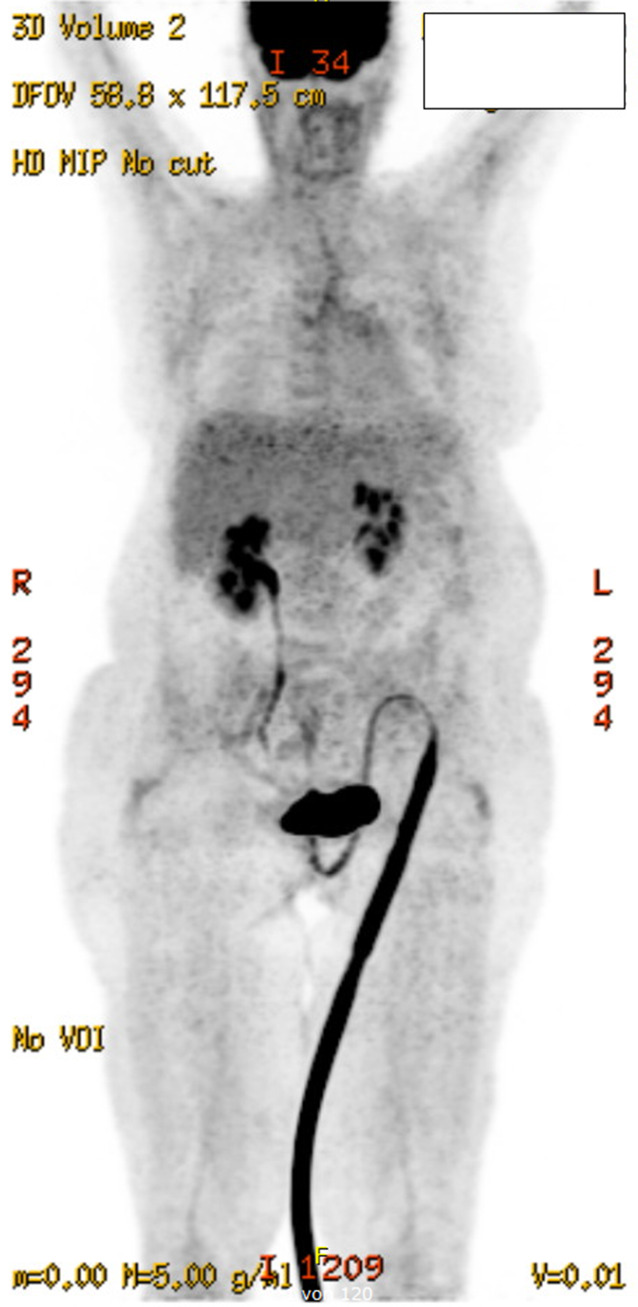
Unauffällige Positronenemissionstomographie/Computertomographie, Fall 2

Wir diagnostizierten eine HLH mit 7/8 positiven HLH-Kriterien, 255 Punkten im HScore (Wahrscheinlichkeit > 99 %) und einem positiven OHI-Index. Als nächsten diagnostischen Schritt veranlassten wir eine PET/CT, die den zugrunde liegenden Auslöser nicht erkennen ließ (Abb. 3), sowie eine Knochenmark- und Leberbiopsie, die aufgrund erhöhter Leberenzyme durchgeführt wurde. Beide Biopsien zeigten eine Hämophagozytose ohne Hinweise auf eine Lymphominfiltration.

**Abb. 4:**
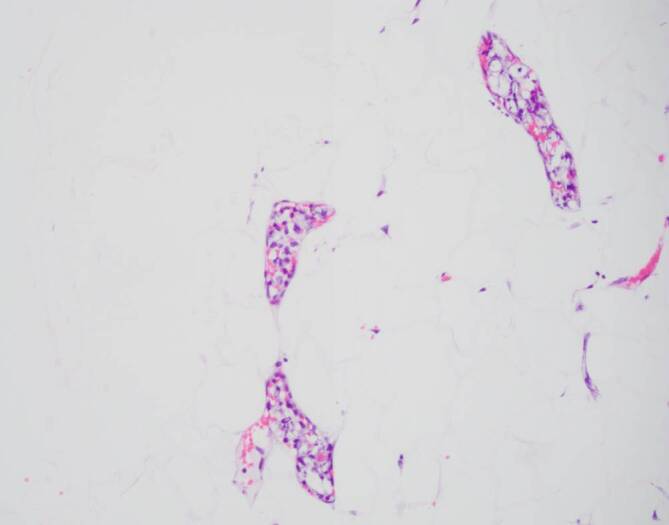
Blutgefäße mit intravaskulären Lymphominfiltraten

Der Zustand des Patienten verschlechterte sich rapide mit Multiorganversagen. Schließlich ergab eine Biopsie der makroskopisch normalen Haut die Diagnose eines intravaskulären DLBCL (Koexpression von CD20 und BCL6) mit hoher Wachstumsfraktion von 80 bis 90 % (Abb. 4 und 5).

**Abb. 5:**
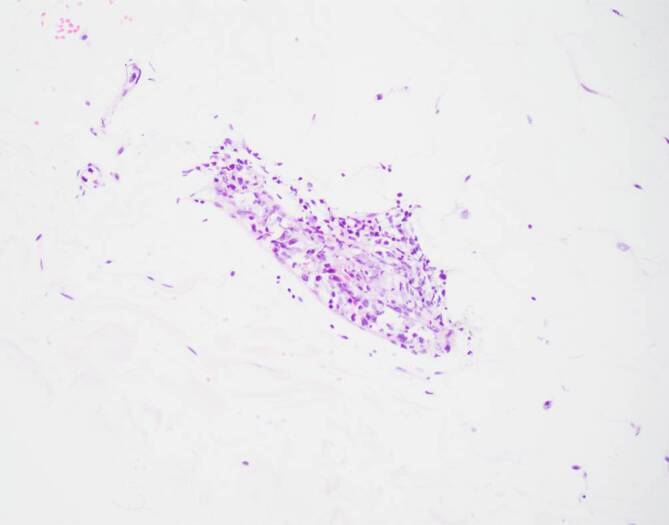
Blutgefäße mit intravaskulären Lymphominfiltraten

Es wurde eine Therapie mit insgesamt 6 Zyklen Rituximab, Cyclophosphamid, Doxorubicin, Vincristin und Prednisolon (R-CHOP) und 2 Zyklen hoch dosiertem Methotrexat eingeleitet, die zu einer vollständigen Remission und einer vollständigen klinischen Erholung führte.

## Diskussion

Die HLH ist eine seltene Komplikation oder Erstpräsentation von DLBCL. Für Deutschland wird die Inzidenzrate einer HLH bei Erwachsenen auf 0,59/100.000 pro Jahr geschätzt [8]. Die Sensitivität der FDG-PET/CT für den Nachweis des zugrunde liegenden Malignoms bei M‑HLH wurde mit 48,9–83 % angegeben [7, 9, 10].

Eine negative PET/CT schließt bei HLH ein Lymphom als Auslöser nicht aus

Die beiden Fälle verdeutlichen jedoch, dass eine negative PET/CT bei HLH ein zugrunde liegendes Lymphom als Auslöser nicht ausschließt. Der OHI-Index war in beiden Fällen stark positiv, was auf ein Lymphom als Grunderkrankung hindeutet. So konnte im ersten Fall auch ohne FDG-Anreicherung im Knochenmark eine hochgradige Lymphominfiltration nachgewiesen werden. Die anderen Lymphommanifestationen waren in beiden Fällen ungewöhnlich. Das intravaskuläre Lymphom im zweiten Fall konnte ebenfalls nicht mittels PET/CT nachgewiesen werden. Daher sollte bei Verdacht auf M‑HLH auch im Falle einer negativen PET/CT eine gründliche Abklärung mit (eventuell wiederholten) Knochenmarkpunktionen sowie Biopsien der Haut und eventuell anderer Organe wie Leber und Milz bis hin zur Splenektomie [11] angestrebt werden.

## Fazit für die Praxis

Bei unklarem Fieber und erhöhten Entzündungsmarkern sollte frühzeitig an eine hämophagozytische Lymphohistiozytose (HLH) gedacht werden. Eine schnelle und rigorose diagnostische Abklärung, einschließlich Knochenmarkpunktion sowie Biopsien der Haut und möglicher anderer Strukturen, ist auch im Falle einer negativen Positronenemissionstomographie/Computertomographie entscheidend, um den zugrunde liegenden Auslöser der HLH finden und eine angemessene Therapie einleiten zu können.

## Data Availability

Die Daten, die die Ergebnisse dieser Studie untermauern, sind auf Anfrage beim Autor erhältlich. Die Daten sind aus Gründen des Datenschutzes oder aus ethischen Gründen nicht öffentlich zugänglich.
